# Dosimetric comparison of the helical tomotherapy, volumetric-modulated arc therapy and fixed-field intensity-modulated radiotherapy for stage IIB-IIIB non-small cell lung cancer

**DOI:** 10.1038/s41598-017-14629-w

**Published:** 2017-11-01

**Authors:** Yujin Xu, Weiye Deng, Shuangyan Yang, Pu Li, Yue Kong, Ye Tian, Zhongxing Liao, Ming Chen

**Affiliations:** 1 Department of Radiation Oncology, The Second Affiliated Hospital of Soochow University, Suzhou, China; 2 Department of Radiation Oncology, Zhejiang Cancer Hospital, Hangzhou, China; 3 Department of Radiation Oncology, The University of Texas, M. D. Anderson Cancer Center, Houston, USA; 4 Department of Radiation Physics, Zhejiang Cancer Hospital, Hangzhou, China

## Abstract

The study aimed to compare the dosimetric parameters to target dose coverage and the critical structures in the treatment planning of helical tomotherapy (TOMO), volumetric-modulated arc therapy (VMAT), and fixed-field intensity-modulated radiotherapy (IMRT) for NSCLC delivering conventionally fractionated radiotherapy. Thirty patients with pathologically confirmed NSCLC were included. Three radiation treatment plans were designed for each patient. All patients received the uniform prescription dose of 60 Gy to the planning target volume. The conformity index (CI), heterogeneity index (HI), and parameters of critical structures were calculated. A significantly superior mean CI was observed in VMAT than in TOMO or IMRT (*P* = 0.013, 0.001). Mean HI was also better using VAMT or IMRT than TOMO (*P* = 0.002, 0.003). Mean lung V_20_ and V_30_ were significantly reduced by TOMO compared to IMRT (*P* = 0.019, 0.029). The heart was spared by IMRT compared to TOMO in terms of mean heart dose, V_5_, V_10_, and V_20_ (*P* < 0.05). In larger tumor, VMAT provided the optimal dose distribution and sparing to heart. Compared to TOMO and IMRT, VMAT achieved better target dose distribution and similar sparing of critical structures. VMAT seemed to be the optimal technique for NSCLC.

## Introduction

Radiation therapy (RT) plays a crucial role in the treatment of non-small cell lung cancer (NSCLC). More modern radiation techniques have appeared with the development of radiation equipment and radiation physics in recent years. It seems particularly critical to choose a most suitable radiation technique for NSCLC patients. Intensity-modulated radiotherapy (IMRT) represents the most popular and advanced RT technique for its better conformity and homogeneity and sparing of organs at risk (OARs) by using non-uniform radiation beam intensities and inverse planning method in NSCLC treatment^1^. Fixed-field IMRT, delivered using linear accelerators fitted with multileaf collimator (MLC), has become the most popular modality of IMRT and is considered as the standard technique of IMRT. It is also referred to as IMRT routinely^2^. Retrospective studies have revealed that IMRT improved the survival outcome and reduced high-grade pneumonitis incidence rate compared to conformal RT^3,4^.

Volumetric-modulated arc radiotherapy (VMAT) is a novel form of IMRT technique and is regarded as a new generation linear accelerator IMRT. Unlike fixed-field IMRT, VMAT deliver intensity modulated radiation beam arcs with simultaneously coordinated gantry rotation, MLC shape and motion, and dose rate modulation^5,6^. In addition, VMAT has been reported to be a better dose conformity or sparing of OARs with a shorter treatment time than IMRT in different solid cancers^7–9^.

Helical tomotherapy (TOMO) is another novel approach of the IMRT techniques using a helical 360° radiation delivery system, similar to a spiral computed tomography (CT) scan. Compared to conventional fixed-field IMRT, TOMO has the advantage of using a higher number of independent beam directions, which may result in better dose conformity to target. By rapid opening and closing of leaves in a collimator rotating around the patient, TOMO provides the ability to sculpt radiation doses to complex shaped tumorous regions while avoiding doses to normal organs^10^. Nowadays, TOMO is frequently used for a variety of diseases^11–14^. However, the clinical value of TOMO in lung cancer is still controversial so far. At the same time, TOMO and VMAT may deliver more extensive low-dose irradiation to the surrounding normal lung tissue. This may potentially be harmful, especially in combination with chemotherapy or target therapy^15–17^.

Although all of these three modern radiation techniques are capable of achieving treatment plans with high conformity while reducing the dose delivered to the surrounding OAR, there is no consensus on the “optimal” treatment technique to NSCLC so far. In this dosimetric study, we explored and compared the dosimetric parameters to target dose coverage and the OARs in the treatment planning of TOMO, VMAT and IMRT for NSCLC delivering conventionally fractionated radiotherapy.

## Material and Methods

### Patient clinical data

From August 2015 to May 2016, a total of 30 patients with pathologically confirmed NSCLC were enrolled in the Department of Radiation Oncology at Zhejiang Cancer Hospital. All the patients were medically inoperable, or they refused to have an operation. The treatment plan was radical radiotherapy or combined chemoradiotherapy. Clinical stage ranged from IIB to IIIB according to the 7^th^ edition of the American Joint Committee on Cancer (AJCC) staging manual for lung cancer. The median age of the 30 patients was 62 years old (range, 40–79 years). Most of them were males (29 patients, 96.7%). The detailed clinical and pathological characteristics of the 30 patients were summarized in Table 1. Informed consent forms were signed by all patients. The ethics institutional review board of Zhejiang Cancer Hospital approved the protocols for data collection and analyses. All the methods described here were performed in accordance with the relevant guidelines and regulations.

**Table 1:** Patient characteristics.

Characteristic		N	%
Sex	Male	29	96.7
	Female	1	3.3
Age (years)	Median	62	
	Range	40–79	
Histology	SCC	17	56.7
	AC	9	30.0
	NSCC-NOS	4	13.3
Primary tumor location	LUL	8	26.7
	LLL	3	10.0
	RUL	12	40.0
	RML	4	13.3
	RLL	3	10.0
Primary tumor size (cm)	Median	3.3	
	Range	1.3–7.8	
T stage	T1	4	13.3
	T2	10	33.3
	T3	7	23.3
	T4	9	30.0
N stage	N0	1	3.3
	N1	6	20.0
	N2	12	40.0
	N3	11	36.7
Clinical TNM stage (AJCC 7^th^)	IIB	2	6.7
	IIIA	15	50.0
	IIIB	13	43.3
	Central	11	36.7
	Peripheral	19	63.3
PTV volume (cm^3^)	Median	312.84	
	Range	89.34–650.44	
Total lung volume (cm^3^)	Median	3512.30	
	Range	2119.45–4938.96	

Abbreviation: SCC = squamous cell carcinoma; AC = adenocarcinoma; NSCC-NOS = non-small cell carcinoma-not otherwise specified; LUL = left upper lobe; LLL = left lower lobe; RUL = right upper lobe; RML = right middle lobe; RLL = right lower lobe; PTV = planning tumor volume.

### Targets delineation and dose prescription

**Figure 1:**
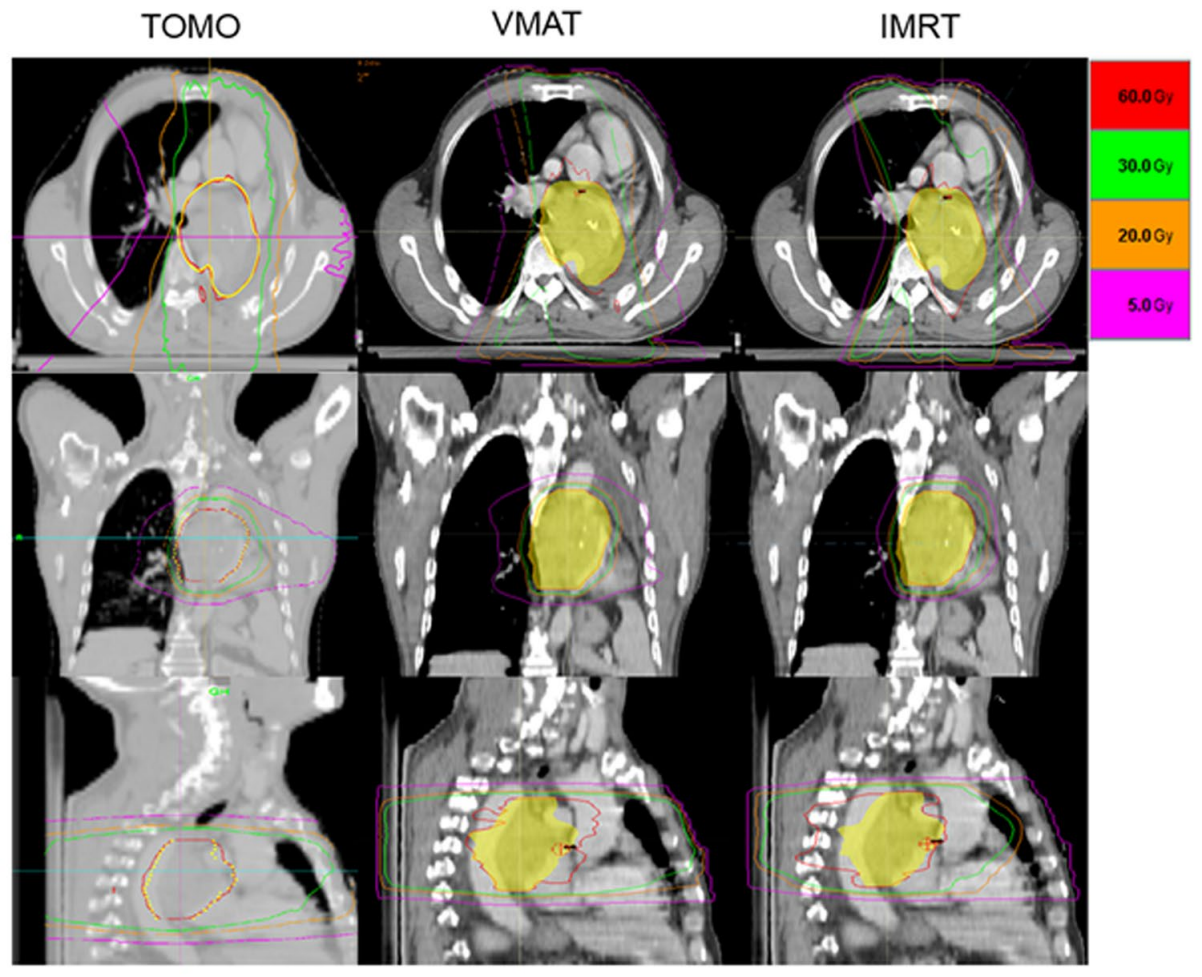
Typical isodose distributions for helical tomotherapy (TOMO), volumetric-modulated arc therapy (VMAT), and intensity-modulated radiotherapy (IMRT) plans for a patient showing the same CT slice. The planning target volume was painted in yellow. The pink, orange, green and red lines represent the dose curves of 5, 20, 30, and 60 (the prescription dose) Gy, respectively.

All the patients underwent four-dimensional computed tomography (4D-CT) with Philips Brilliance CT Big Bore simulator in the supine position and free breathing conditions. Patients were scanned using the bellows device placed around the abdomen. Images were binned in 10 phases, with 5-mm thickness throughout the entire neck, thorax, and upper abdomen. The primary lung tumor and lymph nodes measuring ≥ 1 cm in short-axis diameter on thoracic enhanced CT and/or PET positive intake were included in the gross tumor volume (GTV). The internal GTV was contoured on a reconstructed maximum intensity projection image using the 10-phase 4D-CT simulation scan and verified across all phases of the 4D-CT dataset^18^. The internal clinical target volume (ICTV) was created by expanding the 6–8mm isotropic margin without extending into uninvolved organs. The planning target volume (PTV) was generated by expanding the ICTV by 5 mm isotropically. The TOMO, VMAT, and IMRT treatment plans were performed using Tomotherapy (Accuray Incorporated, Sunnyvale, CA) and Raystation (RaySearch Laboratories AB, Stockholm, Sweden) treatment planning software for each patient. Fixed seven-field and two-arc technique was used in the IMRT and VMAT plans, respectively. A total of 60 Gy in 30 fractions was prescribed to the PTV. The constraints of OARs mainly included as follows: Lung V20 (i.e., percentage of the total lung volume receiving ≥ 20 Gy) ≤ 33%, mean lung dose (MLD) ≤ 17 Gy; mean heart dose (MHD) ≤ 35 Gy, heart V40 ≤ 60%; spinal cord maximum dose ≤ 45 Gy; esophageal maximum dose ≤ 105% of prescription dose. To insure the consistency of all radiation plans, two specially appointed experienced radiation physicians completed and optimized the three different plans of the same patient. All the radiation therapies were performed with linear accelerator 6MV-X. Typical dose distributions for TOMO, VMAT, and IMRT plans of one patient are shown in Fig. 1.

### Treatment plan evaluation

To compare the dosimetric differences among the three modern radiation techniques, the dose–volume histograms for the PTV, total lung, heart, esophagus, and spinal cord were calculated. To evaluate the precise fitting of the radiation distribution to the PTV, the conformity index (CI) was used, which was calculated according to the following equation^19^: CI = (V_ROI, pres_)^2^/(V_ROI_*V_body, pres_), where V_ROI, pres_ is the volume of PTV covered by the prescription dose, V_ROI_ is the volume of PTV, and V_body, pres_ is the total volume covered by the prescription dose. The closer CI value to 1 means the higher conformity of the radiation plans. The heterogeneity index (HI) was defined as^20^: (D_2_-D_98_)/D_pres_, where D_2_ and D_98_ correspond to radiation doses delivered to 2% and 98% of the PTV, respectively. D_pres_ is the prescription dose to PTV. The lower HI value means the better radiation distribution. D_1_, D_2_, D_50_, D_95_, D_98_, D_99_ (D_V_: radiation doses delivered to v% of the PTV) and V_95_, V_100_, V_105_ (V_D_: the percentage volume of PTV receiving D% prescription dose or more) were calculated for each case. The mean dose, V_5_, V_10_, V_20_, V_30_, V_40_ and V_50_ (V_D_:0D Gy or more) of total lung and heart, the mean esophagus dose and maximum dose (D_max_) to the spinal cord and esophagus were recorded as well.

### Statistical analysis

All dosimetric parameters were analyzed by applying “mean ± SD”. Post hoc Student’s t-tests were applied for pair wise comparisons of relevant dosimetric parameters when a one-way analysis of variance (ANOVA) was statistically significant. Statistical analyses were performed using SPSS version 23.0 (SPSS Inc., Chicago, IL, USA). All p-values were two-sided, and a p-value less than 0.05 was considered statistically significant.

## Results

### Target dose coverage

**Figure 2:**
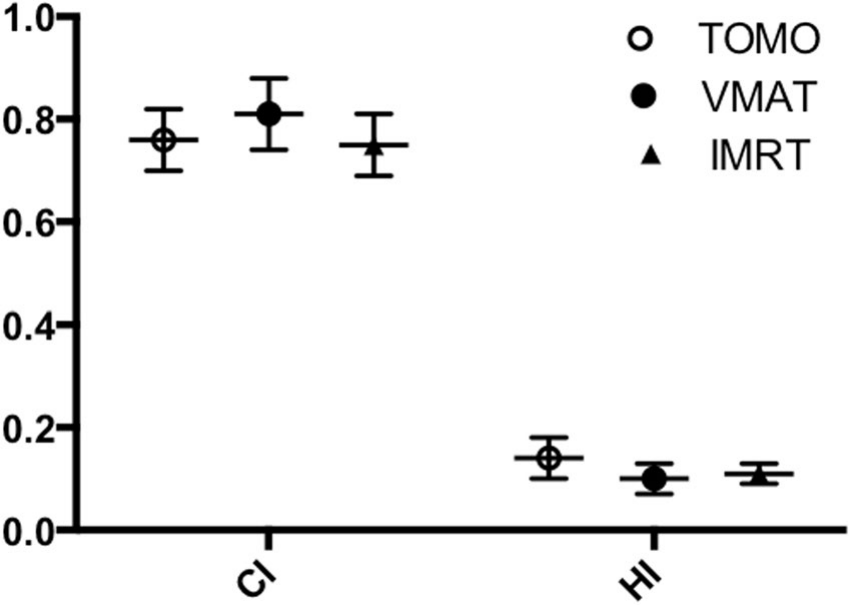
Comparison of conformity index and heterogeneity index of planning target volume among the three radiation techniques.

PTV dosimetric parameters and comparisons among the three radiation techniques were summarized in Table 2. Compared with the other two techniques, VMAT generally provided a higher CI and a lower HI, indicating a more conformal and homogeneous dose distribution to the PTV (Fig. 2). The mean CI was significantly superior by VMAT compared to either TOMO or IMRT techniques (*P* = 0.013, 0.001, respectively). The mean HI was also significantly better by VAMT and IMRT compared to TOMO (*P* = 0.002, 0.003, respectively). The mean dose to PTV by VMAT was 62.41 Gy, which was significantly decreased compared to plans by TOMO (63.37 Gy, *P* < 0.001) and IMRT 62.68 Gy, *P* = 0.047). In terms of high-dose areas (D_1_, D_2_) and low-dose areas (D_98_, D_99_), V_95_, and V_105_, TOMO was significantly inferior compared to the other two techniques (*P* < 0.05), indicating worse dose distribution by the TOMO planning.

**Table 2:** Comparison of target and OARs’ dose-volume parameters in three radiation techniques.

		TOMO	VMAT	IMRT	*P* value		
					T vs. V	T vs. I	V vs. I
PTV	CI	0.76 ± 0.06	0.81 ± 0.07	0.75 ± 0.06	0.013	0.290	0.001
	HI	0.14 ± 0.04	0.10 ± 0.03	0.11 ± 0.02	0.002	0.003	0.070
	D_mean_(Gy)	63.37 ± 1.08	62.41 ± 0.61	62.68 ± 0.66	0.000	0.002	0.047
	D_max_ (Gy)	66.39 ± 1.29	65.40 ± 1.02	65.66 ± 0.93	0.007	0.011	0.203
	D_1_ (Gy)	65.61 ± 1.29	64.67 ± 0.90	64.95 ± 0.81	0.005	0.019	0.134
	D_2_ (Gy)	65.43 ± 1.27	64.23 ± 1.65	64.79 ± 0.79	0.006	0.023	0.103
	D_50_ (Gy)	63.77 ± 1.18	62.60 ± 0.71	62.86 ± 0.54	0.000	0.000	0.062
	D_95_ (Gy)	59.82 ± 0.48	59.94 ± 0.25	59.91 ± 0.23	0.267	0.421	0.616
	D_98_ (Gy)	57.16 ± 1.99	58.41 ± 0.71	58.24 ± 0.61	0.004	0.011	0.265
	D_99_ (Gy)	54.94 ± 3.47	57.05 ± 1.20	56.65 ± 1.16	0.004	0.019	0.163
	V_95_ (%)	98.32 ± 0.89	99.03 ± 0.57	98.88 ± 0.49	0.001	0.006	0.224
	V_100_ (%)	94.63 ± 0.76	94.83 ± 0.65	94.73 ± 0.69	0.252	0.674	0.522
	V_105_ (%)	63.22 ± 19.24	35.05 ± 22.15	43.15 ± 16.45	0.000	0.000	0.054
Total lung	MLD (Gy)	12.64 ± 3.75	12.39 ± 3.07	12.50 ± 3.18	0.834	0.969	0.868
	V_5_ (%)	44.44 ± 12.20	43.43 ± 12.62	42.25 ± 11.11	0.898	0.295	0.615
	V_10_ (%)	32.72 ± 10.08	31.47 ± 8.59	32.86 ± 8.71	0.662	0.519	0.438
	V_20_ (%)	21.80 ± 7.47	22.21 ± 6.01	24.24 ± 6.20	0.762	0.019	0.141
	V_30_ (%)	15.14 ± 5.88	15.74 ± 4.35	16.71 ± 4.07	0.677	0.029	0.324
	V_40_ (%)	10.60 ± 4.75	11.03 ± 3.43	11.32 ± 3.82	0.747	0.157	0.756
	V_50_ (%)	7.07 ± 3.67	7.25 ± 2.76	7.29 ± 3.15	0.872	0.767	0.959
Heart	MHD (Gy)	14.22 ± 8.73	11.69 ± 7.41	11.23 ± 7.06	0.228	0.033	0.882
	V_5_ (%)	51.99 ± 27.95	41.41 ± 25.97	38.91 ± 24.81	0.278	0.002	0.684
	V_10_ (%)	40.00 ± 25.66	30.31 ± 22.11	29.76 ± 20.13	0.231	0.013	0.913
	V_20_ (%)	28.21 ± 21.35	21.80 ± 16.92	21.32 ± 16.17	0.198	0.043	0.792
	V_30_ (%)	19.11 ± 15.17	14.02 ± 11.33	14.61 ± 11.35	0.234	0.067	0.801
	V_40_ (%)	11.25 ± 9.14	9.07 ± 7.89	9.13 ± 7.29	0.485	0.189	0.969
	V_50_ (%)	5.75 ± 5.18	5.19 ± 5.48	4.97 ± 4.50	0.922	0.641	0.842
Spinal cord	D_max_ (Gy)	37.10 ± 10.75	34.82 ± 10.02	37.03 ± 8.84	0.447	0.785	0.352
Esophagus	D_max_ (Gy)	65.37 ± 1.22	60.04 ± 13.31	60.48 ± 11.81	0.351	0.324	0.866
	Mean (Gy)	33.47 ± 2.06	31.98 ± 1.47	32.26 ± 1.68	0.411	0.512	0.776

Abbreviation: OAR = organs at risk; PTV = planning target volume; CI = conformity index; HI = heterogeneity index; T = helical tomotherapy; V = volumetric-modulated arc therapy; I = intensity-modulated radiotherapy; MLD = mean lung dose; MHD = mean heart dose.

### Sparing doses to OARs

**Figure 3:**
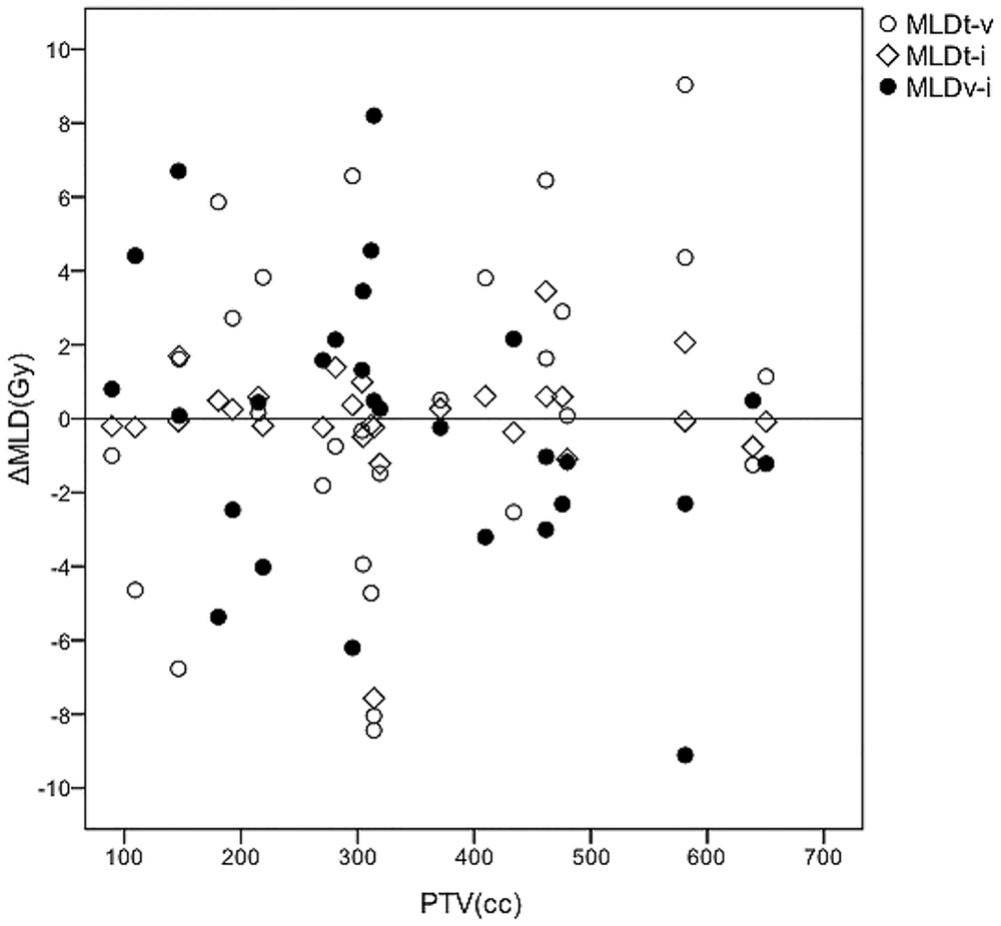
Correlations between PTV and differences among the three radiation techniques in mean lung dose (MLD) for the total lung. ΔMLD stands for the differences between two radiation plans in MLD. For the patients with smaller volumes, the three radiation plans were comparable, but for larger volumes, VMAT showed the better MLD compared with TOMO and IMRT plans in MLD.

**Figure 4:**
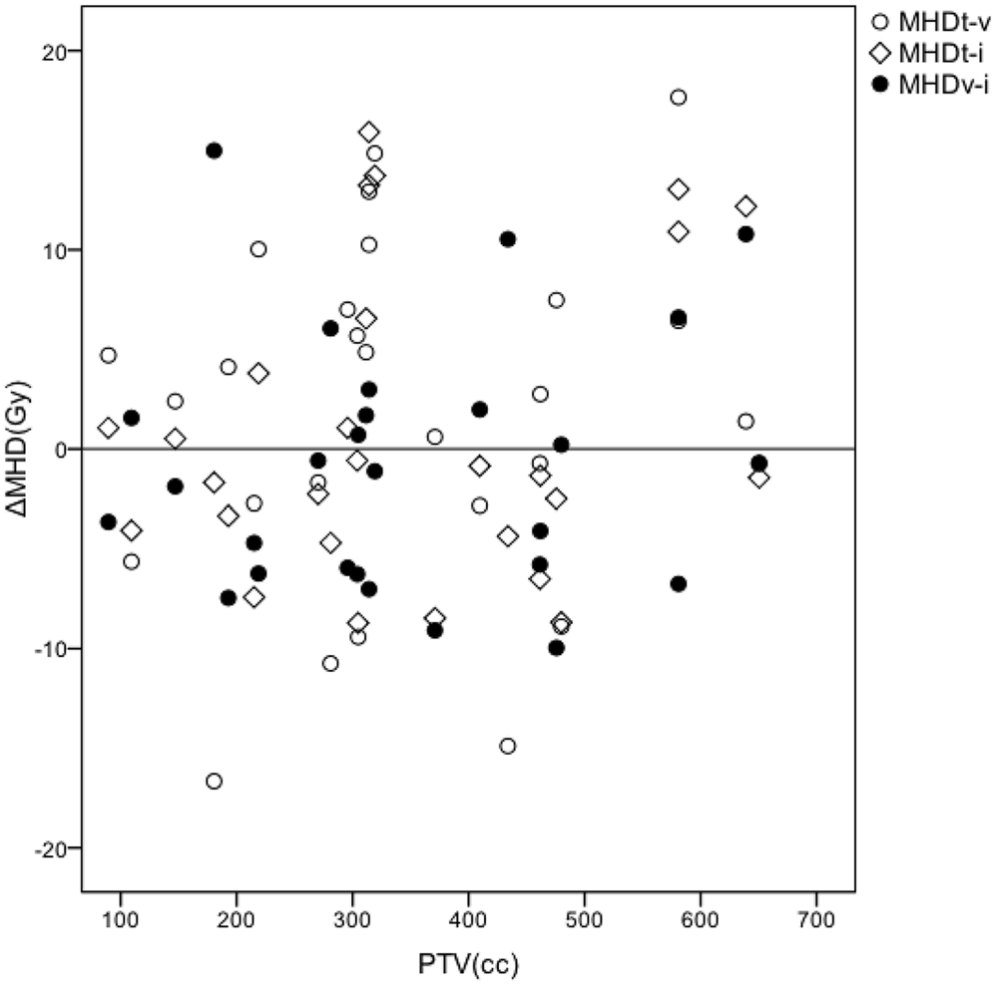
Correlations between PTV and differences among the three radiation techniques in mean heart dose (MHD). ΔMHD stands for the differences between two radiation plans in MHD. For the large volumes, TOMO seems to be inferior to IMRT and VMAT plans in MHD.

The dose parameters of OAR and targets were listed in Table 2. MLD, V_5_, V_10_, V_40_, and V_50_ for the total lung were similar by all three techniques. Mean V_20_ and V_30_ of lung were significantly reduced by the TOMO plan compared to IMRT plan (V_20_: 21.80% vs. 24.24%, *P* = 0.019; V_30_: 15.14% vs. 16.71%, *P* = 0.029). The heart was spared significantly by IMRT plan compared to TOMO plan in terms of MHD, V_5_, V_10_, and V_20_ (*P* < 0.05). The comparative discrepancies of MLD and MHD among the three techniques for each patient were drawn on Figs 3 and 4. The mean esophagus dose and maximum doses to the esophagus and spinal cord were comparable among the three radiation techniques (*P* > 0.05).

### Dosimetric comparison in subgroup analysis

We divided the cohorts into three kinds of subgroups according to the primary tumor type, volume, and location. In the centrally located lung lesions, VMAT also showed a significantly superior CI and HI than the other two techniques in CI and HI. Compared with the IMRT plan, the mean V_20_ of lung was significantly reduced by the TOMO plan (21.06% vs. 23.38%, *P* = 0.002), but V_5_ conversely increased (43.41% vs. 39.12%, *P* = 0.002). While in the peripherally located lung lesions, there were no significant differences in dosimetric parameters delivered to the lung, heart, spinal cord, and esophagus among all three techniques. In comparison to TOMO and IMRT, VMAT had a slight advantage to CI and HI (Table 3). We selected the median PTV volume of 312 mm^3^ as the cutoff value to separate the larger target volume from the smaller target volume. In the subgroup of larger target volumes, VMAT had statistical advantages over CI (*P* = 0.002) to IMRT and HI to TOMO (*P* = 0.034). Meanwhile, VMAT was significantly superior to MHD, V_5_, V_10_, and V_20_ of the heart compared with TOMO (*P* < 0.05). In the smaller target volumes, CI was similar among the three techniques and TOMO provided the worst HI and heart V_5_ (Table 4). Otherwise, in terms of the left-lung tumors, TOMO had better lung sparing than VMAT and IMRT, especially in lung V_20_ and V_30_ compared with IMRT (*P* < 0.05). However, VMAT had significantly superior advantage to heart sparing (MHD, V_10_, V_20_, and V_30_) compared with TOMO in left-lung tumors (*P* < 0.05). As to the right-lung tumors, VMAT indicated the best CI and HI compared to TOMO and IMRT (*P* < 0.05). TOMO had statistically inferior MLD (*P* = 0.037), heart V_5_ (*P* = 0.013), and V_10_ (*P* = 0.037) compared with IMRT (Table 5).

**Table 3:** Comparison of target and OARs’ dose-volume parameters in the subgroup of centrally and peripheral located lung lesions.

			TOMO	VMAT	IMRT	*P* value		
						T vs. V	T vs. I	V vs. I
Central	PTV	CI	0.76 ± 0.07	0.83 ± 0.06	0.76 ± 0.04	0.026	0.969	0.004
		HI	0.14 ± 0.04	0.09 ± 0.02	0.10 ± 0.02	0.001	0.038	0.228
	Lung	V_5_ (%)	43.41 ± 8.69	43.28 ± 13.26	39.12 ± 10.67	0.974	0.002	0.336
		V_20_ (%)	21.06 ± 6.53	22.26 ± 7.48	23.38 ± 5.49	0.695	0.002	0.650
Peripheral	PTV	CI	0.77 ± 0.06	0.79 ± 0.07	0.74 ± 0.07	0.198	0.157	0.034
		HI	0.14 ± 0.05	0.10 ± 0.03	0.11 ± 0.02	0.042	0.036	0.175

Abbreviation: OAR = organs at risk; PTV = planning target volume; CI = conformity index; HI = heterogeneity index; T = helical tomotherapy; V = volumetric-modulated arc therapy; I = intensity-modulated radiotherapy; MLD = mean lung dose.

**Table 4:** Comparison of target and OARs’ dose-volume parameters in the subgroup of larger tumor volume (PTV ≥ 312 mm^3^) and smaller tumor volume **(**PTV < 312 mm^3^).

			TOMO	VMAT	IMRT	*P* value		
						T vs. V	T vs. I	V vs. I
PTV ≥ 312mm^3^	PTV	CI	0.77 ± 0.06	0.81 ± 0.05	0.73 ± 0.07	0.057	0.155	0.002
		HI	0.15 ± 0.05	0.10 ± 0.03	0.12 ± 0.02	0.034	0.083	0.051
	Heart	MHD (Gy)	18.21 ± 9.50	12.76 ± 8.15	12.56 ± 7.24	0.118	0.040	0.527
		V_5_ (%)	63.63 ± 27.74	47.16 ± 27.96	46.99 ± 22.79	0.155	0.032	0.714
		V_10_ (%)	50.96 ± 28.13	35.49 ± 25.31	34.82 ± 20.53	0.146	0.035	0.719
		V_20_ (%)	37.90 ± 24.22	24.82 ± 19.85	24.22 ± 17.83	0.134	0.034	0.631
PTV < 312 mm^3^	PTV	CI	0.76 ± 0.07	0.80 ± 0.08	0.76 ± 0.05	0.122	0.836	0.101
		HI	0.13 ± 0.04	0.10 ± 0.03	0.10 ± 0.01	0.015	0.014	0.877
	Heart	V_5_ (%)	39.46 ± 23.05	38.37 ± 24.90	30.51 ± 22.67	0.900	0.000	0.350

Abbreviation: OAR = organs at risk; PTV = planning target volume; CI = conformity index; HI = heterogeneity index; T = helical tomotherapy; V = volumetric-modulated arc therapy; I = intensity-modulated radiotherapy; MHD = mean heart dose.

**Table 5:** Comparison of target and OARs’ dose-volume parameters in the subgroup of left-lung and right-lung tumors.

			TOMO	VMAT	IMRT	*P* value		
						T vs. V	T vs. I	V vs. I
Left	Lung	V_20_ (%)	17.76 ± 6.94	22.81 ± 6.77	22.98 ± 5.86	0.156	0.028	0.968
		V_30_ (%)	12.23 ± 6.51	15.72 ± 5.02	16.38 ± 4.68	0.208	0.018	0.756
	Heart	MHD (Gy)	17.55 ± 10.16	8.80 ± 5.45	14.90 ± 7.42	0.047	0.196	0.065
		V_10_ (%)	48.63 ± 28.40	23.89 ± 14.43	41.31 ± 22.53	0.048	0.197	0.072
		V_20_ (%)	37.05 ± 25.15	15.00 ± 10.11	28.89 ± 18.52	0.038	0.127	0.066
		V_30_ (%)	26.01 ± 18.21	10.81 ± 8.03	19.57 ± 12.81	0.045	0.094	0.097
Right	PTV	CI	0.74 ± 0.06	0.81 ± 0.05	0.75 ± 0.07	0.000	0.787	0.001
		HI	0.15 ± 0.04	0.09 ± 0.03	0.11 ± 0.02	0.001	0.003	0.048
	Lung	MLD (Gy)	13.56 ± 3.46	12.42 ± 2.83	12.09 ± 3.15	0.188	0.037	0.509
	Heart	V_5_ (%)	47.73 ± 26.84	46.68 ± 28.28	37.08 ± 21.08	0.779	0.013	0.070
		V_10_ (%)	34.93 ± 23.29	34.62 ± 24.97	24.50 ± 16.79	0.718	0.037	0.086

Abbreviation: OAR = organs at risk; PTV = planning target volume; CI = conformity index; HI = heterogeneity index; T = helical tomotherapy; V = volumetric-modulated arc therapy; I = intensity-modulated radiotherapy; MHD = mean heart dose; MLD = mean lung dose.

## Discussion

To the best of our knowledge, the present study is the first report comparing dosimetric parameters of three different modern radiation techniques, which are TOMO, VMAT, and IMRT, in radical radiotherapy for stage IIB-IIIB NSCLC. From the results of the study, we found that the dose coverage, conformity, and homogeneity of the PTV and the sparing of critical structures adjacent to the tumor target were satisfactory in all three plans, but the VMAT technique had a better conformal coverage and dose distribution compared to the TOMO and IMRT techniques. Otherwise, lung V20 and V30 were significantly reduced by TOMO compared to IMRT. Conversely, the heart was spared significantly by the IMRT plans compared to the TOMO plans in terms of MHD, V_5_, V_10_, and V_20_ (*P* < 0.05). The mean maximum doses to the esophagus and spinal cord were comparable among the three radiation techniques (*P* > 0.05).

IMRT has been regarded as the “standard” radiation technique and has been widely used in the clinic^21^. However, the clinical value of TOMO remains controversial in terms of NSCLC, especially in locally advanced lung cancer such as the cases with larger and/or centrally located lesions or for patients who have widespread lymph node involvement cases. Some studies have shown that TOMO can improve target coverage while sparing critical organs compared to fixed-field IMRT in many solid tumors^22–24^. A study by Kron *et al*.^25^ compared TOMO plans with IMRT plans generated using 6 to 10 coplanar beams for 15 patients with stage III inoperable NSCLC. All patients had treatment plans of 60 Gy at the primary target and 46 Gy at the regional lymph nodes, including the mediastinum. A good correlation was found between the quality of the TOMO plans and the IMRT plans with TOMO being slightly better than those of the IMRT in most cases. The overlap between lung and PTV was found to be a good indicator of plan quality for TOMO. For early-stage NSCLC, the TOMO technique performed better dosimetrically as compared to the seven-field coplanar IMRT and the two-arc coplanar RapidArc, reducing maximum rib dose, as well as improving dose conformity and uniformity^26^. The study by Xhaferllari *et al*.^27^ provided an extensive dosimetric planning among fixed-beam IMRT, VMAT, and TOMO for early-stage NSCLC with SABR. The results demonstrated that VMAT had the optimal trade-off in dose conformity, sparing normal tissue, and treatment efficiency when compared with fixed-beam. VMAT outperformed TOMO in all parameters measured and was advantageous in treating early-stage NSCLC with SABR compared to fixed-beam, while providing significantly shorter treatment times. The results were nearly consistent with our findings. In our subgroup analysis, we found that TOMO created the reduction of lung V20 at the cost of increasing V5 spread to normal lung in centrally located lung lesions. Meanwhile, TOMO did not show a significant benefit on target dose coverage. With the comprehensive consideration, the TOMO radiation technique showed an inferior status compared to the VMAT and IMRT. We should be cautious to adopt the TOMO technique in the treatment of locally advanced NSCLC.

VMAT has been reported to create better dose conformity or sparing of OARs with a shorter treatment time than IMRT in treating different cancers^10–15^. Theoretically, the VMAT technique may also produce a large volume of low dose regions in the surrounding normal tissue. Such wide distribution of low dose might be harmful to the patient with regard to lung cancer^18,19^. The results of the present study demonstrated that the VMAT technique generally improved the conformal coverage and dose distribution compared to the TOMO and IMRT techniques. On the other hand, almost all the dosimetric parameters of sparing the surrounding organs were comparable with TOMO and IMRT. Especially, in the subgroup of larger target volume, VMAT provided the optimal technique compared with the other two plans, regardless of dose distribution or sparing the normal heart. As to comprehensive evaluation, VMAT seems to be the optimal treatment among the three techniques for unresectable stage IIB-IIIB NSCLC.

Radiation pneumonitis was one of the most common radiation-related complications for thoracic malignancies, especially for lung cancer. The incidence of radiation pneumonitis was strongly correlated with the radiation dose delivered to the normal lung. A number of studies indicated that the dosimetric parameters from the lung DVH were independent and these significant risk factors were associated with the occurrence of severe radiation pneumonitis^28,29^. Lung V20 and MLD were regarded as the most crucial parameters in our clinic. In our present study, the V20 from TOMO plans have been shown to be decreased when compared with IMRT in the whole cohorts and the subgroups of centrally located lung lesions and left-lung tumors. However, low-dose sparing to the normal lung tissue conversely increased from the intrinsic nature of TOMO radiation delivery. The mean MLD was comparable among three radiation techniques in the whole cohorts and subgroup population.

There were some limitations in this study. First, the comparison conclusions drawn from the study were specific to the three ways of IMRT plans, which were fixed-field IMRT, VMAT, and TOMO techniques. For fixed-field IMRT, a seven-field coplanar arrangement was designed, and for VMAT a two-arc coplanar beam configuration was used. Actually, these modalities could have been planned using more beams or a non-coplanar beam arrangement, which might increase plan complexity and even change the results compared with each other. Second, the parameter evaluation of normal lung tissue generally referred to the total lung, so we did not divide into ipsilateral and contralateral lung as reported in some of the literature. Whether the distinguishing assessment are needed requires further study. In addition, the limited sample size enrolled in this study might cause insufficient statistical power to show significance among some of the dosimetric parameters. More clinical studies with large sample sizes are essential in the future.

## Conclusions

In the treatment of stage IIB-IIIB NSCLC patients with different IMRT techniques, our present study demonstrated that the VMAT plan achieved optimal conformal and homogeneous dose distribution in terms of PTV. TOMO plan showed a slight advantage in reducing the sparing of the total normal lung, mainly in V_20_ and V_30_, but at the cost that more low-dose area spread to the normal lung and more radiation doses to the heart. These findings may be of value in selecting the optimal modality of radiotherapy for the individual patient with LA-NSCLC. Although all three different IMRT plans were clinically acceptable, VMAT seems to be the optimal treatment planning technique in the dosimetric comparison with TOMO and IMRT as to comprehensive evaluation.
